# Chitosan as an Immunomodulating Adjuvant on T-Cells and Antigen-Presenting Cells in Herpes Simplex Virus Type 1 Infection

**DOI:** 10.1155/2016/4374375

**Published:** 2016-12-21

**Authors:** Bunsoon Choi, Do-Hyun Jo, A. K. M. Mostafa Anower, S. M. Shamsul Islam, Seonghyang Sohn

**Affiliations:** ^1^Department of Microbiology, Ajou University School of Medicine, Suwon, Republic of Korea; ^2^Department of Applied Chemistry and Biological Engineering, Ajou University, Suwon, Republic of Korea; ^3^Department of Biomedical Sciences, Ajou University, Suwon, Republic of Korea

## Abstract

Herpes disease caused by herpes simplex virus type 1 (HSV-1) is an intractable condition. It is a major concern in public health. Our purpose of this study was to verify the function of chitosan as an adjuvant for immune regulation specifically under herpes simplex virus type 1 (HSV-1) infection. Ahead of HSV infection, chitosan, heat inactivated green fluorescent protein expressing HSV (G-HSV), and a combination of chitosan and G-HSV were used to pretreat ICR mice followed by HSV-1 infection. Using flow cytometric analysis, the frequencies of T-cells, monocytes, dendritic cells (DCs), and natural killer (NK) cells were analyzed by surface expression of CD4^+^, CD8^+^, CD14^+^, CD11c^+^, NK1.1^+^, and DX5^+^ cells. In HSV infected mice, chitosan treatment significantly increased the frequencies of CD4^+^ T-cells (33.6 ± 5.78%) compared to those in the control group (24.02 ± 12.47%, *p* = 0.05). The frequencies of DC and NK cells were also significantly different between chitosan treated mice and control mice. In addition, anti-HSV IgG antibody was downregulated in chitosan treated mice. These results suggest that chitosan is a potential modulator or immune stimulator as an adjuvant in HSV-1 infected mice.

## 1. Introduction

Chitosan is a biocompatible, biodegradable, and natural nontoxic biopolymer with high cationic potential. It is produced by the deacetylation of chitin, a major component in the shells of crustaceans such as crab, shrimp, and crawfish [1]. Chitosan is a safe and effective adjuvant candidate suitable for a broad spectrum of prophylactic and therapeutic vaccines. Recently, chitosan has received considerable attention for its commercial applications in the biomedical, food, and chemical industries [2–4]. Chitosan exhibits many biological effects, including antimicrobial [5, 6] and hypocholesterolemic activities [7, 8] for drug delivery [9, 10]. Chitosan solution enhances both humoral and cell-mediated immune responses to subcutaneous vaccination [11]. Vaccination with chitosan hydrogel is as effective as a dendritic cell vaccination in tumor protection with more readily detectable immune correlates of protection [12]. Recently, it has been reported that chitosan can modulate immune responses by increasing T-cell, B-cell, monocyte, and macrophage cell markers in normal mice [13]. Several researchers over 20 years ago have found that chitosan could be a potent activator of macrophages and NK (natural killer) cells with immune adjuvant capabilities [14–16].

Herpes simplex virus type 1 (HSV-1) is a common and precarious human pathogen that causes a variety of diseases ranging from mild skin disorders to life-threatening encephalitis. It has been extensively studied in animal models [17–19]. In murine models, HSV-specific CD4 and CD8 T lymphocytes have been shown to play vital roles in controlling primary and recurrent HSV infections [20]. In human recurrent lesions, monocytes and CD4 T lymphocytes infiltrate first followed by CD8 T lymphocytes that appear to clear HSV infection [21–23]. HSV infection of keratinocytes in vitro and in vivo induces the secretion of a sequence of chemokines and cytokines such as IFN-*β*, IL-12, IL-1*β*, and IL-6 [24]. *β* chemokines probably attract monocytes and CD4 and CD8 T lymphocytes into lesions. IFN-*α*/*β* and IL-12 may entrain Th1 patterns of cytokine response from HSV antigen stimulated CD4 and CD8 T lymphocytes [25]. Recently, the importance of a distinct immunological synapse between NK, DC, and CD4 T-cells was reported in herpetic skin lesions [26]. From these results, DC and NK cells can be considered as targets for HSV vaccine development. In our previous results, treatment with an oral chitosan-pCIN-mIL-4 mixture was found to lead to expression of IL-4 mRNA and protein in intestinal tissues and increased serum levels of IL-4 in mice. It has been reported that chitosan encapsulated pDNA enables effective transfer of GFP gene into cells in vivo [27].

In this study, we investigated the role of chitosan as an immune-stimulatory or immune-modulatory adjuvant in HSV-1 infection by analyzing the frequencies of antigen-presenting cells (APCs) in LN and peripheral blood mononuclear cells (PBMC) of normal mice.

## 2. Materials and Methods

### 2.1. Mice and Experimental Groups

In this study, 4- to 5-week-old ICR male mice were used. Animals were handled in accordance with a protocol approved by the animal care committee of the Ajou University School of Medicine (AMC-102, Suwon, Republic of Korea).

### 2.2. Preparation of Heat Inactivated GFP-HSV

Green fluorescent protein incorporated herpes simplex virus (GFP-HSV) was a gift from Professor Yasushi Kawaguchi [28]. GFP-HSV stock was propagated in monolayer cultures of Vero cells overlain with minimum essential medium (MEM) supplemented with 10% bovine serum and antibiotics. GFP-HSV was inactivated at 65°C for 30 min in an incubator. The inactivation was confirmed by further culture. Heat inactivated green fluorescent protein expressing HSV (G-HSV) was mixed with 200 mL PBS and orally administered three times at ten-day interval.

### 2.3. Preparation and Administration of Chitosan

Chitosan was prepared from chitin of red crabs (*Chionoecetes japonicus*) by treating with NaOH. For the isolation of 50 KDa chitosan, size exclusion chromatography (SEC) and ultrafiltration method were applied [29]. The eluent fraction of SEC was lyophilized after desalting and redissolved in distilled water for the filtration through 0.45 *μ*m membrane filter and, then, applied to ultrafiltration membrane installed in ultrafiltration cell (Amicon 8400, Millipore, USA). Filtered materials were freeze dried, solubilized in distilled water, precipitated in acetone/ether (50/50, V/V) mix, and vacuum dried (10^−3 ^torr). Chitosan powder was dissolved in 1% acetic acid (chitosan acetate) (0.35% w/v) and administered orally three times at 10-day interval either alone or with G-HSV. Acetic acid 1% was used as a control throughout this study.

### 2.4. HSV-1 Infection In Vivo

Virus inoculation was performed using published procedure [30]. Briefly, the earlobes of mice were scratched with a needle and inoculated with 1 × 10^6^ plaque-forming units (p.f.u.)/mL of HSV-1 (F strain) that had been grown in Vero cells. Virus inoculation was performed twice with a 10-day interval. For the virus inoculation, the mice were euthanized by intramuscular injection in the hind leg with ketamine/xylazine cocktail (15 mg/kg ketamine and 10 mg/kg xylazine). Chitosan was administered before HSV inoculation. Chitosan (Chit), heat inactivated GFP-HSV (G-HSV), or chitosan with G-HSV (Chit+G-HSV) was administrated orally into normal mice for three times at 10-day interval. On the next day following the second inoculation, the mice were sacrificed. For HSV infection, 10 days after the last chitosan administration, HSV was inoculated. The next day after the 2nd HSV inoculation, mice were used for further experiments. PBMC and lymph node cells were isolated for analysis of cell frequencies using flow cytometry. A figure representing the frequencies of cells positive for indicated markers was prepared. The numbers of mice used in each group were marked in figures and figure legends.

### 2.5. Flow Cytometry

PBMC and LN cells were isolated from mice and washed with PBS, after which 1 × 10^6^ cells were incubated at 4°C for 30 min with the following antibodies: FITC anti-mouse CD4, PerCP-Cy5.5 anti-mouse CD8, PE-Cy7 anti-mouse CD3, APC anti-mouse CD14, APC anti-mouse CD11c, FITC anti-mouse CD11b, PE anti-mouse NK1.1, FITC anti-mouse DX5, PE anti-mouse IL-7R, and APC anti-mouse IL-15Ra (eBioscience, Inc., USA). Cells were analyzed using a flow cytometer (FACSCanto II, Becton Dickinson, San Jose, CA, USA).

### 2.6. Measurement of HSV-1 IgG by ELISA

After sacrificing the mice by dislocation of cervical vertebrae, blood was collected from heart and serum was analyzed using a commercial ELISA kit for IgG levels of mouse HSV-1 (Mouse-Rat HSV-1 IgG ELISA kit, GenWay Biotech Inc., CA, USA). ELISA was conducted according to the manufacturer's instructions. The absorbance values of samples were read at a wavelength of 450 nm using a Bio-Rad model 170-6850 microplate reader.

### 2.7. Statistical Analysis

All data are represented as the mean ± SD. Statistical differences between experimental groups were determined by Student's *t*-test and Bonferroni correction. Statistical analysis was conducted using MedCalc® version 9.3.0.0. Statistical significance was considered when *p* value was less than 0.05.

## 3. Results

### 3.1. Effect of Chitosan on Proliferation of T Lymphocytes in Lymph Node and PBMC without or with HSV Infection

**Figure 1:**
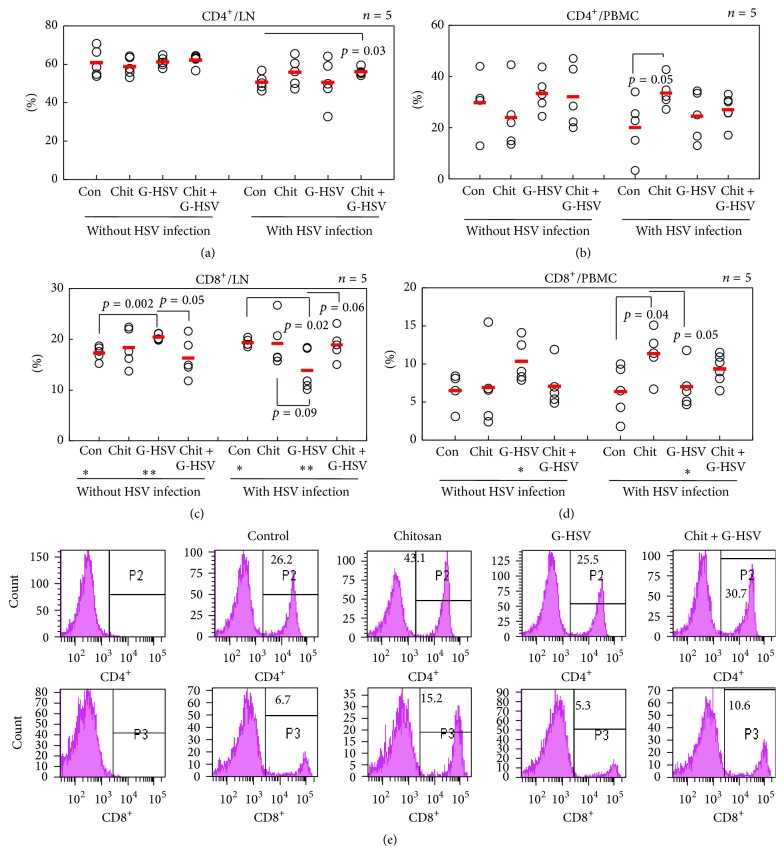
Efficacy of chitosan on CD4^+^ and CD8^+^ T-cells with or without HSV infection. Administration of 1% acetic acid (control), 0.23% chitosan (Chit), heat inactivated GFP-HSV (G-HSV), or chitosan with G-HSV (Chit+G-HSV) orally into normal mice for three times at 10-day intervals. On the next day, mice were sacrificed or inoculated with HSV-1 at 10 days later as described in the Materials and Methods. Isolated PBMC and lymph node cells were applied for analysis of CD4^+^ and CD8^+^ T-cells by flow cytometry (a–d). The frequencies of CD4^+^ and CD8^+^ T-cells in PBMC were significantly upregulated in chitosan treated mice compared to those in control mice infected with HSV. (e) Representative histogram of the frequencies of CD4^+^ and CD8^+^ cells in HSV infected PBMC. The number of mice used in each group was five. In (c) ^*∗*^ *p* = 0.01 and ^*∗∗*^ *p* = 0.004 and in (d) ^*∗*^ *p* = 0.05.

To evaluate the effect of chitosan on proliferation of T lymphocytes, we administered chitosan, G-HSV, or chitosan with G-HSV orally into normal mice for three times at 10-day intervals. On the next day after the final administration, mice were sacrificed or infected with HSV after another 10 days as described in the Materials and Methods. On the next day after HSV inoculation, PBMC and lymph node cells were isolated and analyzed by flow cytometry. The frequencies of CD4^+^ T-cells in lymph nodes in HSV infected mice (50.76 ± 4.03%, 55.90 ± 7.38, 50.66 ± 12.06%, and 56.16 ± 1.97%, resp.) were lower than those in mice without infection (60.86 ± 7.39%, 58.90 ± 4.80%, 61.26 ± 2.63%, and 62.16 ± 3.11%, resp.) (Figure 1(a)). Although the frequencies of CD4^+^ T-cells in lymph nodes of infected mice showed lower expressions, they were not significantly different between chitosan and the combination of chitosan with G-HSV (Figure 1(a)). In PBMC of HSV infected mice, the frequencies of CD4^+^ T-cells in chitosan treated mice (33.6 ± 5.78%) were significantly higher (*p* = 0.05) than those of control group (24.02 ± 12.47%) (Figure 1(b)). However, the frequencies of CD4^+^ T-cells in mice treated with G-HSV or the combination of chitosan and G-HSV (24.52 ± 9.64%, 27.08 ± 5.66%, resp.) were lower than those in the uninfected group (33.40 ± 7.21% or 32.16 ± 12.21%).

Under the same treatment conditions, with respect to the proportions of CD8^+^ T-cells in lymph nodes of HSV infected groups, G-HSV treated group expressed a lower (*p* = 0.004) proportion compared to uninfected group (20.44 ± 0.65% versus 13.88 ± 4.08%, *p* = 0.004, Figure 1(c)). Without HSV infection, G-HSV treatment upregulated (*p* = 0.002) the frequencies of CD8^+^ T-cells compared to the control. The combination treatment with chitosan and G-HSV downregulated (*p* = 0.05) the frequencies of CD8^+^ T-cells compared to the G-HSV. In HSV infection, G-HSV treatment downregulated (*p* = 0.02) the frequencies of CD8^+^ T-cells compared to the control while the combination treatment of chitosan and G-HSV recovered the frequencies of CD8^+^ T-cells to control level (G-HSV versus chitosan + G-HSV, *p* = 0.06). In PBMC of mice without HSV infection, the patterns of CD8^+^ T-cell frequencies were similar to those in LN. In mice with HSV infection, chitosan treatment upregulated (*p* = 0.04) the frequencies of CD8^+^ T-cells compared to the control (Figure 1(d)). The representative FACS histograms of the frequencies of CD4^+^ and CD8^+^ cells in HSV infected group appear in Figure 1(e).

### 3.2. Correlation of IL-7R and IL-15R*α* Expression in Lymph Node and PBMC of HSV Infected Mice

**Figure 2:**
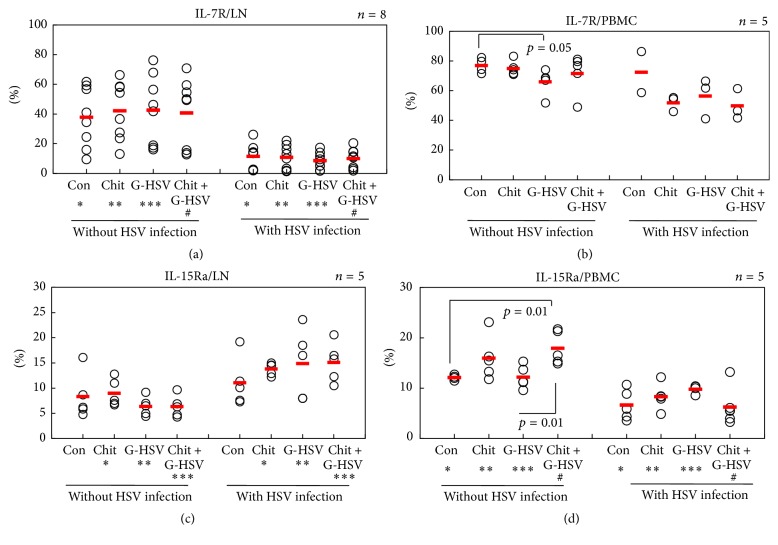
Correlation of IL-7R expression and IL-15R*α* expression in lymph node and PBMC of HSV infected mice. The frequencies of IL-7R^+^ cells in LN were significantly downregulated in all four groups with HSV infection compared to those in mice without HSV infection. The frequencies of IL-15R^+^ cells were significantly different between mice with HSV infection and those without HSV infection. The numbers of mice used in (a) were 8 and in (b), (c), and (d) were 5 in each group. In (a) ^*∗*^ *p* = 0.02, ^*∗∗*^ *p* = 0.0005, ^*∗∗∗*^ *p* = 0.005, and ^#^ *p* = 0.001. In (c) ^*∗*^ *p* = 0.003, ^*∗∗*^ *p* = 0.01, and ^*∗∗∗*^ *p* = 0.001. In (d) ^*∗*^ *p* = 0.005, ^*∗∗*^ *p* = 0.005, ^*∗∗∗*^ *p* = 0.05, and ^#^ *p* = 0.0002.

The IL-15/IL-15R*α* on cell surfaces enables sustained IL-15 activity and contributes to the long survival of CD8 memory T-cells [31]. In addition, loss of IL-7R and IL-15R expression is associated with the disappearance of memory T-cells in respiratory tract following influenza infection [32]. In our experiment, to assess the relevance of IL-7R and IL-15R*α* to chitosan treatment isolated PBMC and LN from HSV infected or uninfected mice were subjected to FACS analysis under the same conditions. Results are shown in Figure 1. In LN with HSV infection, the frequencies of IL-7R cells were not significantly different among groups. In LN without HSV infection, the frequencies of IL-7R cells were not significantly different among groups either. However, the frequencies of IL-7R cells in the four groups with HSV infection were significantly lower compared to those without HSV infection (Figure 2(a)). In PBMC, the frequencies of IL-7R cells in HSV infected groups were decreased compared to those in mice without HSV infection (Figure 2(b)). However, such decreases were not statistically significant.

The frequencies of IL-15R*α* cells in LN were significantly upregulated in HSV infected mice compared to those in mice without HSV infection. However, the difference in frequencies of IL-15R*α* cells between chitosan and the combination of chitosan and G-HSV was not significant (Figure 2(c)). In PBMC, the frequencies of IL-15R*α* cells were significantly downregulated in HSV infected group compared to those in mice without HSV infection (Figure 2(d)). Without HSV infection, the combination of chitosan and G-HSV increased (*p* = 0.01) IL-15R*α* ^+^ cell frequencies compared to G-HSV alone.

### 3.3. Chitosan Has No Influence on the Frequencies of CD14

**Figure 3:**
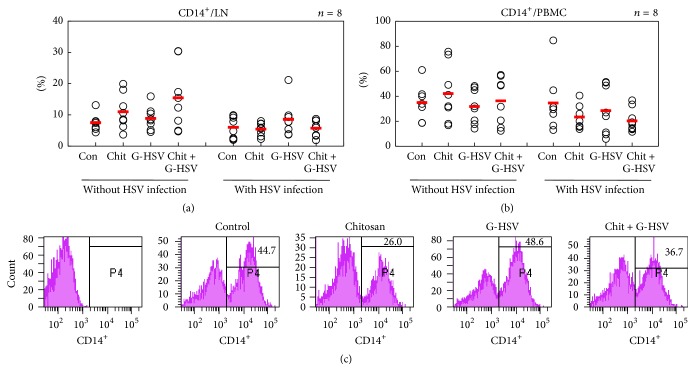
Chitosan has no influence on the frequencies of CD14^+^ monocytes. Chitosan did not significantly regulate the frequencies of CD14^+^ cells in LN or PBMC of HSV infected mice or mice without HSV infection. (c) is the representative histogram showing the frequency of CD14^+^ cells in PBMC with HSV infection. The numbers of mice used were 8 in each group.

CD14 is a Glycosylphosphatidylinositol (GPI) surface anchored molecule particularly expressed on monocytes and macrophages [33, 34]. It plays a role in host defense against respiratory tract infection by influenza A virus [35]. Several reports have shown that CD14 acts as a negative regulator in T-cell activation [36, 37]. To determine the role of chitosan on monocyte and macrophages in HSV infection, CD14 expression was analyzed. Chitosan did not significantly regulate the frequencies of CD14^+^ cells in LN and PBMC in HSV infected mice or mice without HSV infection (Figures 3(a) and 3(b)). CD14^+^ cell population was not significantly influenced by chitosan either, regardless of HSV infection.  Figure 3(c) is representing the FACS histogram frequencies of CD14^+^ cells in PBMC.

### 3.4. Dendritic Cells (DCs) Are Significantly Downregulated in PBMC after HSV Infection

**Figure 4:**
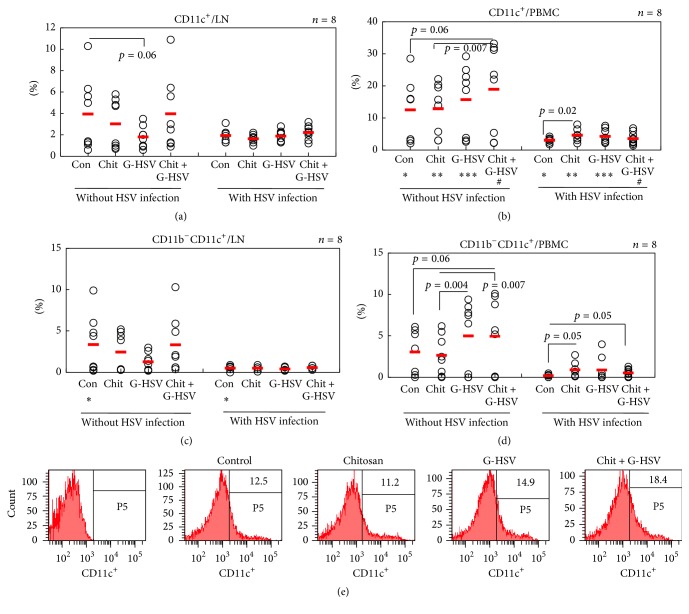
Chitosan controls the frequencies of CD11c^+^ dendritic cells in HSV infection. Chitosan treatment significantly affected the frequencies of CD11c^+^ dendritic cells in HSV infection group. (e) Representative histogram of the frequency of CD11c^+^ cells in PBMC with HSV infection. The numbers of mice used were 8 in each group. In (b) ^*∗*^ *p* = 0.01, ^*∗∗*^ *p* = 0.01, ^*∗∗∗*^ *p* = 0.005, and ^#^ *p* = 0.002. In (c) ^*∗*^ *p* = 0.04.

DCs are rapidly differentiating cells. They are able to capture and process antigen and migrate to lymphoid sites to present antigen to T-cells, thereby inducing adaptive immunity [38]. CD11c is a major marker for identification of DC [39, 40]. In a murine model, it has been demonstrated that the mechanism behind HSV-1 exploitation of DC may involve CD11c [41]. Therefore, we observed the involvement of DCs in this study. In lymph nodes, the frequencies of CD11c^+^ cells were lower in infected mice either in the control group (1.95 ± 0.6%) or in group treated with chitosan alone (1.65 ± 0.38%) or in group treated with the combination of chitosan and G-HSV (2.22 ± 0.66%) compared to those in the uninfected group (3.95 ± 3.43%, 3.03 ± 2.28%, and 3.97 ± 3.46%, resp.) (Figure 4(a)). However, similar expression of CD11c^+^ cells was observed after treatment with G-HSV alone in both infected mice (1.88 ± 0.54%) and uninfected mice (1.81 ± 1.00%). Similar patterns of expression of CD11c^+^ cells were also confirmed in PBMC. All treated mice with HSV infection showed significantly lower expression of CD11c^+^ (3.11 ± 0.76% in control, 4.67 ± 2.01% in chitosan, 4.32 ± 2.19% in G-HSV, and 3.63 ± 1.85% in chitosan with G-HSV) compared to uninfected mice (12.58 ± 10.08%, 12.91 ± 7.93%, 15.76 ± 10.89%, and 18.96 ± 13.58%, resp., Figure 4(b)). In PBMC of HSV infection, chitosan significantly upregulated CD11c^+^ cells compared to the control (*p* = 0.02). The frequencies of CD11b^−^CD11c^+^ expression showed similar patterns of CD11c^+^ frequencies in LN and PBMC of HSV infected mice (Figures 4(c) and 4(d)).

### 3.5. The Frequencies of Natural Killer Cells Are Significantly Increased in Lymph Nodes and PBMC after Infection with HSV

**Figure 5:**
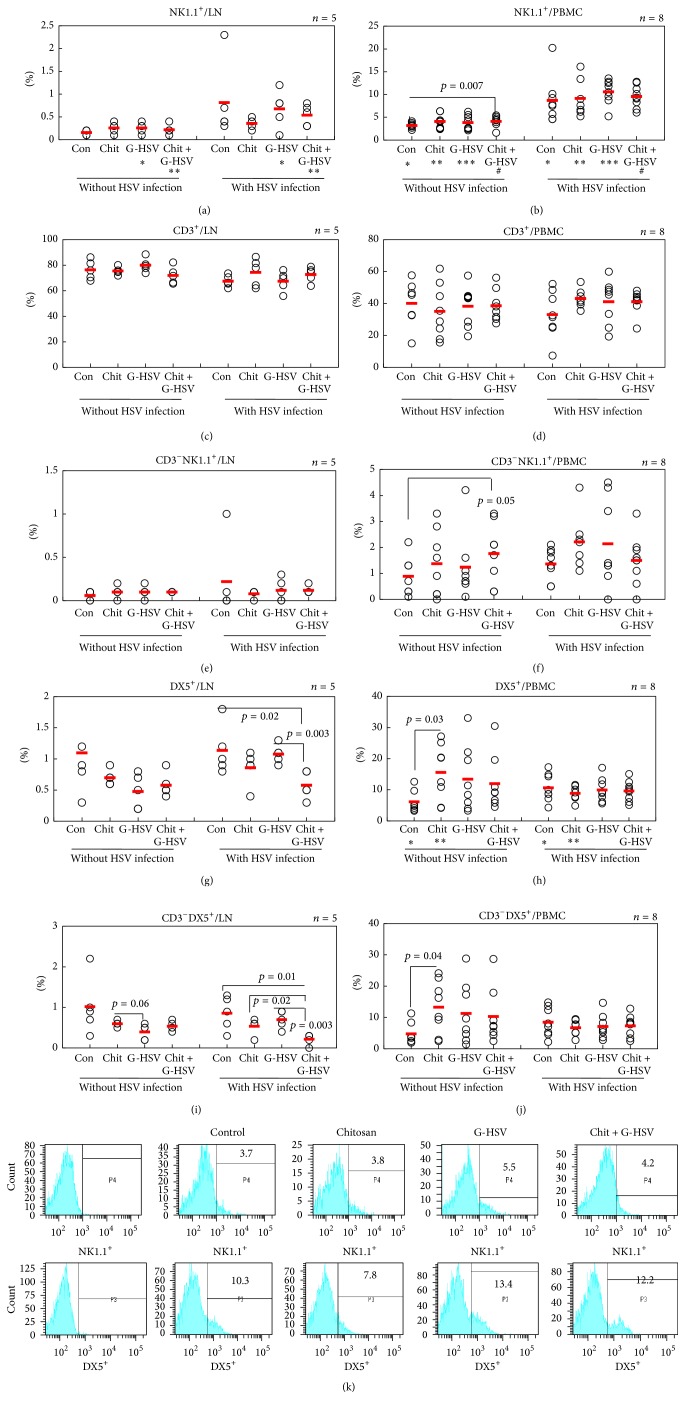
Effect of chitosan on the frequencies of NK cells. The frequencies of NK1.1 were significantly different between mice with HSV infection and those without HSV infection. Chitosan influenced the frequencies of CD3^−^NK1.1^+^ cells in PBMC of mice infected with and CD3^−^DX^+^ cells in PBMC of mice without HSV infection. (k) shows the frequency of NK1.1^+^ and DX5^+^ cells, respectively, in PBMC with HSV infection. The numbers of mice used were 5 in lymph nodes of each group and 8 in PBMC of each group. In (a) ^*∗*^ *p* = 0.03 and ^*∗∗*^ *p* = 0.01. In (b) ^*∗*^ *p* = 0.005, ^*∗∗*^ *p* = 0.003, ^*∗∗∗*^ *p* = 0.00002, and ^#^ *p* = 0.00002. In (h) ^*∗*^ *p* = 0.03 and ^*∗∗*^ *p* = 0.04.

Natural killer (NK) cells have a critical role in the early phases of immune responses against various types of pathogens [42, 43]. The initial stage of HSV-1 infection is influenced by the activity of type 1 interferon, macrophages, and NK cells that can limit early virus replication and spread [44]. We investigated whether NK cells could be generated by the stimulation with chitosan after infection with HSV-1. Although NK cell associated marker NK1.1 expression was not observed markedly in lymph nodes of infected group or uninfected group, the frequencies of NK1.1^+^ cells were significantly higher in G-HSV and treatment with both chitosan and G-HSV in the HSV infected groups compared to those in HSV uninfected group (Figure 5(a)). In PBMC, NK1.1^+^ cells were highly expressed in all infected groups (control, chitosan, G-HSV, or chitosan with G-HSV) (8.71 ± 5.06% in the control, 9.15 ± 4.07% in chitosan, 10.57 ± 2.72% in G-HSV, and 9.56 ± 2.38% in chitosan with G-HSV) compared to those in the uninfected group (3.18 ± 0.63%, 4.07 ± 1.56%, 3.85 ± 1.59%, and 4.11 ± 1.18%, resp., Figure 5(b)). Chitosan combined with G-HSV upregulated (*p* = 0.007) NK1.1^+^ cells compared to the control group without HSV infection (Figure 5(b)). The proportions of CD3 in lymph nodes or PBMC were not significantly different among groups with or without HSV infection (Figures 5(c) and 5(d)). The population of CD3^−^NK1.1^+^ cells was also analyzed in PBMC and lymph nodes. In PBMC, CD3^−^NK1.1^+^ cells were modestly increased in groups with HSV infection (1.36 ± 0.06%, 2.21 ± 1.04%, 2.13 ± 1.68%, and 1.50 ± 0.92%, resp., in control, chitosan, G-HSV, and chitosan with G-HSV) compared to that without infection (0.88 ± 0.75%, 1.37 ± 1.25%, 1.23 ± 1.27%, and 1.76 ± 1.15%, resp., Figure 5(f)) except for those in the group treated with the combination of chitosan and G-HSV (Figure 5(f)). In lymph nodes, there was no remarkable difference in CD3^−^NK1.1^+^ expression (Figure 5(e)). For further confirmation of the involvement of NK cells in this experiment, DX5 was additionally analyzed in PBMC and lymph nodes. DX5, another NK marker, has been useful for identifying NK cells [45]. In PBMC without HSV infection, chitosan significantly (*p* = 0.03) upregulated DX5^+^ cells compared to the control group (Figure 5(h)). In chitosan treated group, DX5^+^ cells were downregulated in mice with HSV infection compared to those in mice without HSV infection. In lymph nodes of HSV infected group, DX5^+^ cells were downregulated in mice treated with the combination of chitosan and G-HSV compared to control mice or mice treated with chitosan alone (Figure 5(g)). There was no distinct difference in the frequencies of DX5^+^ cells between mice with HSV infection and those without HSV infection (Figure 5(g)). CD3^−^DX5^+^ NK cell frequencies were also analyzed. In PBMC of mice without HSV infection, chitosan significantly upregulated CD3^−^DX5^+^ cells compared to the control (Figure 5(j)). There was no significant difference in CD3^−^DX5^+^ NK cell frequencies between mice with HSV infection and mice without HSV infection (with HSV infection: 8.51 ± 4.62%, 6.75 ± 2.45%, 7.16 ± 3.85%, and 7.36 ± 3.27%, resp.; without HSV infection: 4.80 ± 3.62%, 13.30 ± 8.37%, 11.28 ± 9.66%, and 10.30 ± 8.75%, resp., Figure 5(j)). A similar expression pattern of CD3^−^DX5^+^ was observed in LN (Figure 5(i)). With HSV infection, chitosan with G-HSV group significantly downregulated CD3^−^DX5^+^ cells compared to the control, chitosan, or G-HSV treated group (Figure 5(i)). FACS histogram frequencies of NK cells in PBMC are shown in Figure 5(k).

### 3.6. Chitosan Has a Protective Role during Early Infection with HSV-1

**Figure 6:**
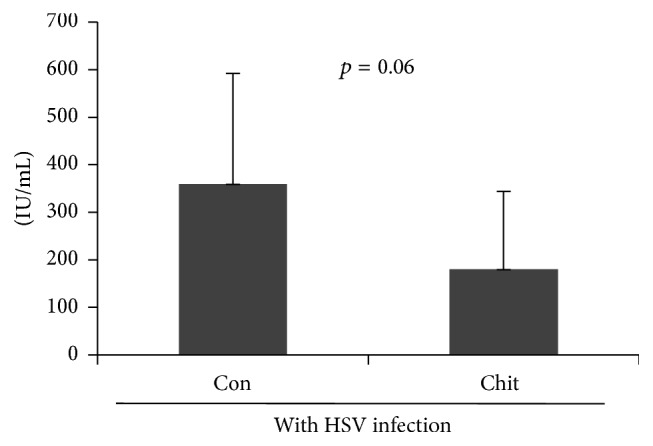
Anti-HSV antibody in chitosan treated and HSV infected mice. The levels of HSV antibody in chitosan treated group were analyzed by ELISA.

To determine the protective role of chitosan against HSV-1 infection, serum IgG levels of HSV-1 in control and chitosan treated mice in HSV infection group were measured by ELISA. Several recent studies have demonstrated that a significant increase in HSV IgG is an indicator of reactivation of or current or recent infection [46, 47]. In this study, production of HSV-1 IgG was downregulated (*p* = 0.06) in chitosan group (180.43 ± 163.58 IU/mL) compared to that in the control group with HSV infection (359.81 ± 232.64 IU/mL, Figure 6). This result suggests that chitosan has a protective effect against HSV-1 infection.

## 4. Discussion 

The innate immune defenses are activated immediately after infection. They are more rapid than specific responses. The elevated innate defenses could delay the onset of disease symptoms, giving the specific immune defenses enough time to develop a more durable protection. Immunostimulants can change the immune status of animals to improve their ability to defend a disease. A recent study has shown the antiviral activity of chitosan against foot and mouth disease virus where chitosan strongly modulates the functional activity of auxiliary cells involved in immune responses such as macrophages and granulocytes [48].

Herpes simplex virus infection may develop immunity in the form of circulating antibody and cell-mediated immunity including T helper, T cytotoxic, and memory T lymphocytes in humans and animals [49, 50]. Moreover, HSV-1 can become latent in the infected host and undergo reactivation, causing recurrent disease even though the host may have intact, innate, and acquired immune defenses [51, 52]. UV-inactivated HSV-1 is incapable of transcribing its early genes, leading to an inability of the virus to replicate [53]. In addition, we have confirmed that chitosan-pcDNA-EGFP-mIL4 DNA vector mixture can improve HSV-1 induced Behcet's disease-like symptoms by increasing IL-4 cytokine [27].

We treated normal mice with chitosan alone, heat inactivated green fluorescent protein incorporated HSV (HI GFP- HSV), or chitosan with HI GFP-HSV to stimulate PBMC and LN cells in vivo before infection with HSV-1. In this study, we first differentiated the effect of chitosan on antigen-presenting cells of PBMC and lymph node during early infection with HSV-1. In our study, chitosan increased CD4^+^ and CD8^+^ T-cell proliferation in PBMC compared to the control in the presence of HSV-1 (Figures 1(b) and 1(d)). This result showed that chitosan could increase the proliferation of CD4^+^ and CD8^+^ T-cells during infection with HSV-1.

Cellular contacts play an important role in the stimulation of T-cells by antigen-presenting cells including monocytes. The presentation of IL-15 by IL-15R*α* on the membrane of activated monocytes is an important mechanism that allows the survival and proliferation of CD8^+^ T-cells [54]. However, IL-7R and IL- 15R expression is gradually lost from persisting memory CD8 T-cells in the airways but not in the spleen [32]. In this report, we found differences in IL-7R and IL-15R expression by lymph node and PBMC during HSV-1 infection. IL-7R was expressed highly in PBMC, but IL-15Ra was increased in the lymph node during early infection (Figure 2).

The CD14 expression is downregulated but monocyte survival is upregulated in human herpes virus type 6 (HHV-6) infection [55]. Recent studies have confirmed the involvement of CD14 in antiviral immunity to control HIV, respiratory syncytial virus, and dengue virus infection [56]. These data strongly support our results. In the presence of HSV-1, CD14 expression was reduced in both PBMC and lymph node compared to that in mice without infection in all treated groups (Figure 3).

DCs are thought to be the most effective antigen-presenting cells (APCs) in immune response with CD11c as a major marker for the identification of DCs [39, 40]. CD11c also controls HSV-1 responses to limit virus replication during primary infection [41]. Furthermore, chitosan nanoparticles have been reported to have immune-stimulating activity such as increasing accumulation and activation of macrophage and polymorphonuclear cell, promoting resistance to infections by microorganisms, and inducing cytokines [57]. Our present results are consistent with these results in that, during early infection with HSV-1, the expression of CD11c in PBMC or lymph node was lower compared to that in uninfected mice (Figures 4(a) and 4(b)). CD11b^−^CD11c^+^ expression in the above same conditions showed a similar pattern as CD11c. Interestingly, mice treated with chitosan alone showed higher expression of CD11c in PBMC with infection compared to that in the control. In contrast, in the absence of infection, chitosan alone could not promote higher expression of CD11c in PBMC, whereas combined treatment of chitosan and GFP-HSV showed higher expression of CD11c compared to chitosan (Figure 4(b)).

NK cells play a decisive role in the optimal clearance of HSV-1 infection in mice. NK1.1 is a marker of natural killer (NK) cells and an alloantigen whose expression is limited to NK cells in several mouse strains [58, 59]. In this study, our result showed higher population of NK1.1^+^ cells in HSV infection groups than that in mice without infection. Chitosan also increased NK1.1^+^ cells in mice without HSV infection. Therefore, chitosan can be used as an adjuvant of NK cell proliferation. DX5 is another marker of NK cells. The increase of DX5 was similar to that of NK1.1 expression. Therefore, chitosan can be a proliferator of NK cells.

In conclusion, we confirmed the role of chitosan as an adjuvant for the immune-stimulatory and immune-modulatory function in HSV-1 infection. Chitosan oral administration amplified the generation of CD11c^+^ DC, NK1.1^+^ NK cells, and DX5^+^ NK cells as well as CD4^+^ and CD8^+^ cells after infection by HSV-1. Our findings showed that chitosan has immunoregulatory functions among antigen-presenting cells, NK cells, and T-cells. We found a complicated involvement of chitosan in mice with or without HSV-1 infection in vivo. These results indicate that chitosan itself can be a potential modulator or immune stimulator in PBMC and lymph nodes during infection with HSV-1.
